# Evaluation of Minnesota Phosphorus Loss Index performance

**DOI:** 10.1002/jeq2.20635

**Published:** 2024-10-08

**Authors:** Heidi Reitmeier, Lindsay Pease, Peyton Loss, Tim Radatz

**Affiliations:** ^1^ Department of Soil, Water, and Climate, Northwest Research & Outreach Center University of Minnesota, Twin Cities Crookston Minnesota USA; ^2^ Minnesota Discovery Farms, Minnesota Agricultural Water Resources Center Eagan Minnesota USA

## Abstract

Supported by the National Phosphorus (P) Research Project led by Dr. Andrew Sharpley, Minnesota developed its statewide P‐Index, the Minnesota P Loss Index (MNPI), to manage critical source areas of agricultural P. The MNPI has remained unchanged since its last revision in 2006. The overall goal of this study was to critically evaluate the MNPI to determine, in the parlance of Sharpley, if the MNPI remains “directionally and magnitudinally correct.” Observed P loss from 67 site‐years of annual edge‐of‐field data was compared with MNPI‐predicted P loss. Our assessment indicates that MNPI performance is directionally correct: it correctly ranks fields that are more at risk than others. The MNPI performed better in years with high‐intensity rainfall events. Averaging MNPI assessment across multiple years of data input, along with minor adjustments to the calculation algorithm, improved the robustness of MNPI estimates. Continued re‐evaluation of the MNPI will ensure that this important tool for nutrient management is properly evaluating P loss potential. This study reflects Dr. Sharpley's decades‐long effort to improve and revise P indices so that they reflect advances in the science and management of agricultural P.

## Core Ideas

- The 2006 Minnesota P Loss Index is directionally correct and can rank sites by P loss potential.
- Average annual soluble P is adequately modeled, but particulate P is overpredicted.
- Model evaluation metrics indicate minor adjustments to improve performance.
- Sharpley's research guided model improvements such as differentiating between transport and source risk.

**Abbreviations**
HABs: harmful algal blooms
MNPI: Minnesota P Loss Index
NSE: Nash‐Sutcliffe efficiency
PBIAS: percent bias
RSR: root mean standard deviation ratio

## 1 INTRODUCTION

Phosphorus loss assessment tools are one way to gauge whether farm management practices align with water quality goals. The early development of the Minnesota P Loss Index (MNPI) followed closely behind efforts to develop P indices in other states. This process is described in detail by Osmond et al. (2024). An initial tabular version of the MNPI was adapted from the original P Index by Lemunyon and Gilbert (1993). Birr and Mulla (2001) determined that Minnesota's tabular version performed well at the watershed scale, but they did not specifically test whether the MNPI could predict P loss at the field scale. In the early 2000s, new guidance on P indices was developed as part of the National P Research Project (Sharpley et al., 2002; Sharpley & Kleinman, 2003). Using this guidance, the MNPI was redeveloped into a weighted and additive model (Birr, 2005; Moncrief et al., 2006). Following this update, work on the MNPI halted for nearly 20 years. Meanwhile, the P research community has continued re‐evaluation and advancement of P indices across North America (e.g., Sharpley et al., 2012, 2017).

The MNPI has not been reviewed or re‐evaluated since its last update in 2006. Since that time, high‐profile freshwater harmful algal blooms (HABs) have increased public attention on reducing P loss from agricultural landscapes (Daloğlu et al., 2012; Schindler et al., 2012). Locally, Minnesota farmers face similar pressure. HABs in Minnesota lakes have been linked to human illness and pet deaths (Heiskary et al., 2014; Lindon & Heiskary, 2009). Availability of edge‐of‐field P loss data have improved, furthering our understanding of how management affects P loss (e.g., Daniels et al., 2018). Subsurface drainage and its transport of soluble P loss are now better understood as significant contributors to edge‐of‐field soluble P loss (e.g., King et al., 2015; Macrae et al., 2007). As an unintended consequence, increased soluble P loss has been linked to agricultural conservation practices such as reduced tillage (e.g., Jarvie et al., 2017).

This study aims to determine whether the MNPI remains “directionally and magnitudinally correct” (Sharpley et al., 2012) considering the many advancements in P research. Thus, the objectives of this study were to (1) use edge‐of‐field monitoring data collected from across the state to compare projected with observed losses, (2) evaluate the relationship between the MNPI‐modeled P loss and the magnitude of losses observed on each farm, and (3) recommend updates to the MNPI to improve its ability to assess P loss potential.

## 2 MATERIALS AND METHODS

### 2.1 Overview and basic components of the MNPI

The MNPI is a weighted and additive model that generates an indicator of P loss potential by considering three forms of edge‐of‐field P loss: sediment‐bound P in rainfall runoff (particulate P); soluble P in rainfall runoff (soluble P); and soluble P in snowmelt runoff (snowmelt P). Detailed descriptions of MNPI inputs, calculations, and references are present in Table 1. Using these equations, a modeled P loss is calculated and reported for each crop year and as an overall average across multiple years of a crop rotation. MNPI‐modeled P loss is then assigned inferred levels of P loss risk as follows: “very low P loss risk” = 0–1 kg ha^−1^; “low P loss risk”: 1–2 kg ha^−1^; “medium P loss risk”: 2–4 kg ha^−1^; “high P loss risk”: 4–6 kg ha^−1^; “very high P loss risk”: 6 kg ha^−1^ or more.

**TABLE 1:** Detailed descriptions of calculations used in the 2006 Minnesota P Loss Index.

Components	Description	Reference
**Particulate P**	**Soil erosion potential** × **manure factor** × **weighted sediment delivery factor** × **soil total P concentration**	
Soil erosion potential	Calculated with revised universal soil loss Equation 2	USDA‐NRCS (2003)
Manure factor	For injected/incorporated manure: Runoff reduced by 25% the year of application, by 16.5% after 1 year, by 8% after 2 years	Gilley and Risse (2000)
Weighted sediment delivery factor	(Fraction of field draining to depressions × “depression constant”) + (Fraction of the field not draining to depressions x smaller value of “sediment delivery ratio” and “sediment trap constant”)	Moncreif et al. (2006)
Depression constant	Depression with no outlet: 0.05; Rock/gravel inlet structures: 0.15; Standard surface inlet: 0.2	Ginting, et al. (2000); Gieseke (2000); Ranaivoson (2004)
Sediment delivery ratio	Proportion of sediment leaving the field that reaches surface water = L^−0.2069^	Ouyang and Bartholic (1997)
	where L is the distance from the field edge to nearest surface water in feet	
Sediment trap constant	Impoundment with runoff storage: 0.05; Water and sediment control basin: 0.2; Terraces: 0.4; Buffer strip: 0.5; None: 1	Iowa NRCS (2001)
Soil total P concentration	Concentration of total P associated with delivered sediment based on soil organic matter and soil test P:	Moncreif et al. (2006)
	Total *p* = 0.6322 + 0.00497 × Olsen‐P + 0.0725 × soil organic matter	
	Bray to Olsen conversion: Olsen‐*p* = 0.7117 × Bray‐P	
	Mehlich to Olsen conversion: Olsen‐*p* = 0.653 × Mehlich‐P	
Estimated soil test P	(Last known soil test *p* value) + [(P_2_O_5_ applied since last soil test—average crop removal) × soil P Buffer factor]	Moncreif et al. (2006)
Average crop removal	30 lb P_2_O_5_ ac^−1^ year^−1^ × years since last soil test; 0 if no crop was harvested	Randall et al. (1997)
Soil P buffer factor	Sandy loam or coarser‐textured soils: 0.05; Finer‐textured soils: 0.03; Calcareous soils (pH > 7.3): 0.02	Moncrief and Evans (1994); Peck et al. (1971)
**Soluble P, rainfall**	**Base runoff volume** × **runoff adjustment factor** × **(soluble soil P + non‐winter applied P)** × **0.22**	
Base runoff volume	Derived from daily precipitation data from 1961 through 1990 for the long‐term average frost‐free dates (−4.4°C base) and the NRCS runoff calculation method assuming soil group B and row crop production (Isoline map in Moncrief et al., 2006)	USDA‐NRCS (1990); Moncreif et al. (2006)
Runoff adjustment factor	Conversion factor to adapt the base runoff volume to other hydrologic groups and cover conditions (table in Moncrief et al., 2006)	Moncreif et al. (2006)
Soluble Soil P	0.0106 × Olsen‐P	Moncreif et al. (2006)
Non‐winter applied P	Surface‐applied P × Incorporation Factor × Surface‐available P	
Surface‐applied P	Total P_2_O_5_ applied during the non‐winter season (Apr. 1 to Nov. 14)	
Incorporation Factor	Constant for incorporation method within 1 week of application (table in Moncrief et al., 2006)	Moncreif et al. (2006)
Surface‐available P	Constant estimating concentration of available P per unit P_2_O_5_ applied: 0.044	Edwards and Daniel (1994); Edwards and Daniel (1992); Mueller et al. (1983)
Unit conversion constant	0.22	
**Soluble P, snowmelt**	**Snowmelt runoff rate x**×**Fall soil condition factor** × **(Fall residue P + Winter‐applied P)** × **Snowmelt‐P**	
Snowmelt runoff rate	65% of the average maximum snowpack for the period March 16 through March 31 (Isoline map in Moncrief et al., 2006)	Hansen et al. (2000); Munyankusi (1999)
Fall soil condition factor	Adjustment for snowmelt retained based on the type and direction of fall tillage (table in Moncrief et al., 2006)	Ginting et al. (1998); Hansen et al. (2000); Munyankusi (1999)
Fall residue P	Surface‐available P based on crop, crop yield, and tillage practice (table in Moncrief et al., 2006)	Halsey (1986); Wischmeier (1973); Hanway and Olsen (1980); Mays et al. (1980)
Winter‐applied P	Total P applied during the winter season (Nov. 15 to Mar. 31)	
Snowmelt‐P	Percent of surface available P lost per unit of snowmelt runoff: 0.18	Moncreif et al. (2006)

### 2.2 Edge‐of‐field monitoring data

**FIGURE 1:**
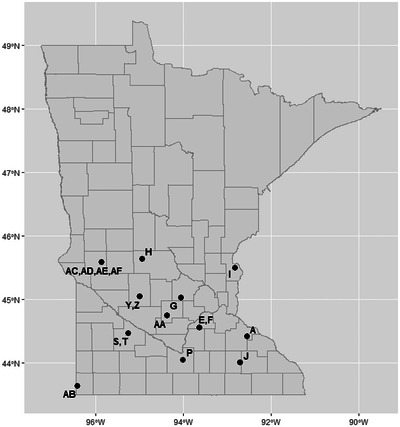
Locations of study sites used in evaluating performance of the Minnesota Phosphorus Loss Index. Abbreviations and information for the study sites are provided in Table S3.

Annual field‐scale edge‐of‐field monitoring data and accompanying management information were requested from researchers and state agencies across Minnesota for potential inclusion in this study. Available data for inclusion in this study were limited. As recently as Christianson et al. (2016), no peer‐reviewed datasets on P loss from subsurface drainage were available within the state of Minnesota. Selection criteria for inclusion were as follows: (1) 1 or more years of annual edge‐of‐field total P loss data; (2) discharge and concentration data available for all relevant pathways (i.e., both surface and subsurface discharge for subsurface‐drained sites); and (3) available management data accompanying years monitored. We identified two privately owned fields with four site‐years (Hansen, 1998), four university research plots with 12 site‐years (Gessel et al., 2004), two privately owned fields with 10 site‐years (Ghane et al., 2016), and 10 privately owned fields with 41 site‐years monitored by Minnesota Discovery Farms (Table S1). Sites were geographically dispersed but were all located in the southern half of the state (Figure 1).

All studies followed standard and comparable methodology for discharge monitoring and sample collection (Table S2). All studies used standard variations of the ascorbic acid method to determine molybdate reactive P (Murphy & Riley, 1962). For all studies, soluble P and total P loss were calculated by multiplying discharge volume by soluble P and total P concentrations, respectively. Particulate P loss was calculated by subtracting soluble P loss from total P loss. Notably, soluble P was not reported by Ghane et al. (2016), so comparisons between predicted and observed P fractions (soluble and particulate P) could not be evaluated for the 10 site‐years observed at the two locations included in this manuscript. These 10 site‐years were excluded from comparisons of observed to modeled loss of soluble P and particulate P.

### 2.3 Evaluation of MNPI performance

Farm management data from each site was entered by crop year into the MNPI. Inputs included P fertilizer application data (source, rate, timing, and placement); soil test P; tillage intensity and timing; crop rotation information; and ground cover at planting (Table S3). This generated an annual MNPI prediction for total P loss, particulate P loss, and soluble P loss (soluble P in rainfall added to soluble P in snowmelt). Although the MNPI only predicts P loss via surface runoff, for the purposes of this evaluation we considered its output to be a prediction of overall edge‐of‐field P loss potential (inclusive of P in subsurface drainage discharge where present) because that is how the MNPI is currently used within the state of Minnesota. The observed annual data could not be separated into “snowmelt” and “rainfall” loss because we did not have access to the original sub‐annual (monthly, weekly, or daily) datasets for most sites. Thus, snowmelt total P loss predicted by MNPI could not be independently verified. To generate a prediction of discharge, we summed the runoff factors “base runoff volume (in) multiplied by runoff adjustment” and “snowmelt runoff rate.” This allowed us to compare MNPI predictions to observed annual discharge.

Following modeling performance evaluation guidelines as described by Moriasi et al. (2007), evaluation of MNPI‐generated P loss potential was compared with observed edge‐of‐field P loss in two ways. First, standard regression to describe how well MNPI predictions fit our observed data and whether the MNPI was directionally correct (i.e., whether higher predictions correlated well with higher observed losses). Second, we evaluated if predictions were magnitudinally correct (i.e., whether the data is in the correct relative range with minimal deviation) using three model evaluation metrics as recommended by Moriasi et al. (2007). These metrics included Nash‐Sutcliffe efficiency (NSE), percent bias (PBIAS), and root mean standard deviation ratio (RSR). Moriasi et al. (2007) reported overall model simulation to be satisfactory if NSE > 0.50, RSR < 0.70, and PBIAS within ± 70%. A similar approach was employed by Good et al. (2012) when evaluating the Wisconsin P Index. All statistical analyses were conducted in JMP 17.0.0 (JMP Statistical Discovery, 2022).

**Core Ideas**

- The 2006 Minnesota P Loss Index is directionally correct and can rank sites by P loss potential.
- Average annual soluble P is adequately modeled, but particulate P is overpredicted.
- Model evaluation metrics indicate minor adjustments to improve performance.
- Sharpley's research guided model improvements such as differentiating between transport and source risk.

## 3 RESULTS AND DISCUSSION

### 3.1 Annual comparison of observed to MNPI‐modeled phosphorus loss

**FIGURE 2:**
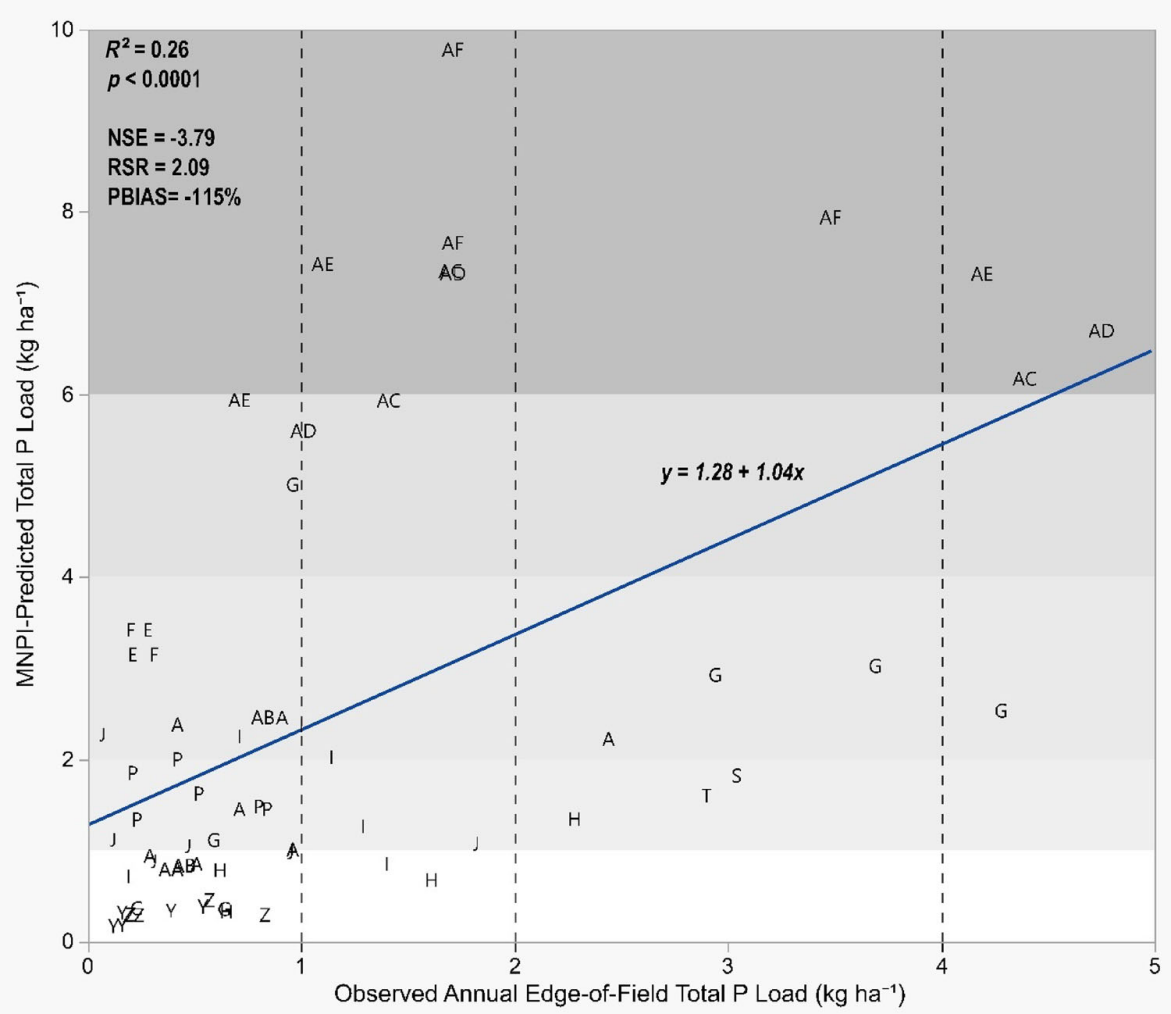
Relationship between MNPI‐predicted and observed annual total P loss. Shaded areas represent assigned risk categories based on modeled P loss (“very low risk of P loss”: 0–1 kg ha^−1^; “low risk of P loss”: 1–2 kg ha^−1^; “medium risk of P loss”: 2–4 kg ha^−1^; “high risk of P loss”: 4–6 kg ha^−1^; “very high risk of P loss”: 6 kg ha^−1^ or more). Dashed vertical lines represent equivalent assigned risk category based on observed data. “NSE” is to Nash‐Sutcliffe efficiency, “RSR” is root mean standard deviation ratio, and “PBIAS” is percent bias as defined in Moriasi et al. (2007). MNPI, Minnesota P Loss Index.

Annual observed edge‐of‐field total P loss was significantly correlated to MNPI‐modeled P loss predictions. Yet model performance metrics indicated poor agreement between predicted and observed P loss overall (Figure 2). Nash‐Sutcliffe model efficiency and PBIAS were both negative. A negative NSE signifies that variance among predicted values is greater than the variance of the measured dataset. Negative PBIAS indicates an overprediction of total P loss. Root mean square error ratio was >1, which indicates a high root mean square error relative to the standard deviation of the dataset (Moriasi et al., 2007). Risk assignments based on MNPI‐modeled P loss and observed P loss agreed in 26 of 67 site‐years (39%). Where assigned risk categories disagreed, assigned risk was greater from MNPI‐modeled P loss than observed P loss in 35 site‐years (52%), and lesser in 6 site‐years (9%).

Similarly, both observed and MNPI‐modeled particulate P loss and observed and MNPI‐modeled soluble P loss had significant positive correlations while performing poorly overall by model performance metrics. Particulate P was overpredicted by the MNPI (PBIAS = −154%, Figure S1). Soluble P tended to be under‐predicted by the MNPI but was closer in magnitude to the observed values than particulate P or total P (PBIAS = 29.3%, Figure S2). This suggests that poor prediction of particulate P may be a limiting factor in MNPI performance. Like many state P indices, MNPI relies on the Revised Universal Soil Loss Equation (RUSLE2) for particulate P estimation. Sharpley et al. (2017) reported that erosion estimation when using RUSLE2 is a common source of overestimation error in predicting P loss.

In general, MNPI performance was directionally correct on an annual basis for each P prediction estimate (total, particulate, and soluble). It was not magnitudinally correct. Other studies have found that empirically‐based models such as the MNPI tend to underperform relative to observed data due to the limitation in their prediction algorithms accounting for annual management changes but not for annual weather variability (Bolster et al., 2017; Good et al., 2012; Sharpley et al., 2017).

To test the degree to which poor hydrologic estimation was contributing to poor model performance, we isolated the component of the model responsible for runoff estimation. Comparing the MNPI's predicted discharge to observed edge‐of‐field discharge revealed runoff prediction to be a key weakness of the model. Predicted discharge was significantly, but weakly, correlated to observed annual edge‐of‐field discharge (*R* ^2 ^= 0.14; *p* = 0.0021). The correlations were not significant between both predicted and observed surface runoff (*R* ^2 ^= 0; *p* = 0.97) and predicted and observed subsurface discharge (*R* ^2 ^= 0.01; *p* = 0.59). Varying weather patterns and site hydrology can greatly affect P transport. Greater P losses are often associated with higher intensity rainfall events (e.g., Gburek & Sharpley, 1998; King et al., 2015). MNPI cannot account for interannual rainfall variability because it uses only static predictions of runoff. Like other annual‐timestep P loss models, no mechanism within the MNPI directly accounts for the complex and time‐dependent processes that control P loss (Bolster et al., 2017; Good et al., 2012).

The MNPI is ultimately intended to be an indicator of P loss potential rather than a predictive model of annual loss. Thus, we evaluated MNPI performance during “worst‐case scenario” years (i.e., years with high‐intensity runoff‐generating events) based on the observation that periods of high cumulative rainfall are often reflected in seasonal and annual measurements of edge‐of‐field discharge and P loss (Pease et al., 2018). We used the historical rainfall record to isolate site‐years with at least 1 day of (1) more than 51 mm of rainfall recorded within a 24‐h period (46 site‐years) and (2) more than 102 mm of rainfall within a 24‐h period (9 site‐years).

Isolating site‐years with higher intensity rainfall events improved regression statistics and model performance metrics relative to the full dataset. Performance metrics for MNPI predictions of TP loss remained in the “unacceptable” range for the 51‐mm 24‐h rain event dataset (Figure S3A). In contrast, the dataset for 102‐mm 24‐h rain events resulted in performance metrics that met “satisfactory” model requirements (Figure S3B). This indicates some reliability in the performance of the MNPI under a “worst‐case scenario” (years with a 102‐mm 24‐h rain event). As discussed above, while the MNPI cannot account directly for the time‐dependent processes affecting P loss, it may indirectly account for them by skewing P loss predictions toward what is observed in higher intensity years. At the same time, the currently available edge‐of‐field dataset is skewed in the other direction. Only 9 site‐years had a 102‐mm 24‐h rainfall event. Further edge‐of‐field data collection during higher rainfall years is needed to add confidence to this observation. Confounding and skewing are common limitations within edge‐of‐field datasets. Continued data collection is critical for further model refinement (Harmel et al., 2008; Sharpley et al., 2017).

### 3.2 Average annual P loss potential

Correlations between both MNPI‐predicted and observed edge‐of‐field P loss improved for total, particulate, and soluble P when across more than one site‐year of data (Table 2). This supports the recommendation that P loss assessment tools should be used for estimating annual average P loss rather than annual P loss (Good et al., 2012; Sharpley et al., 2013; Williams et al., 2016). Despite the improved performance, metrics still fell generally within the “unacceptable range.” Only performance metrics for mean annual soluble P were within an acceptable range. This indicates poor prediction of inter‐annual variability can at least be partially overcome by averaging across years. Lack of improvement in prediction of particulate P provides further evidence that poor soil erosion estimates by RUSLE2 could be influencing poor performance overall.

**TABLE 2:** Model statistics and performance metrics for comparing Minnesota P Loss Index (MNPI) Minnesota P Loss Index (‐predicted annual average P loss to observed annual average P loss values (averages are across all available years by site).

	Linear regression equation	*R* ^2^	*p*‐value	Nash‐Sutcliffe efficiency	Root mean standard deviation ratio	Percent bias (%)
Total P	*y* = 1.34*x* + 1.06	0.28	0.0239	−6.09	2.66	−114
Particulate P	*y* = 2.20*x* + 0.31	0.47	0.0035	−9.34	3.22	−151
Soluble P	*y* = 0.75*x* + 0.03	0.68	<0.0001	0.64	0.60	19.0

**FIGURE 3:**
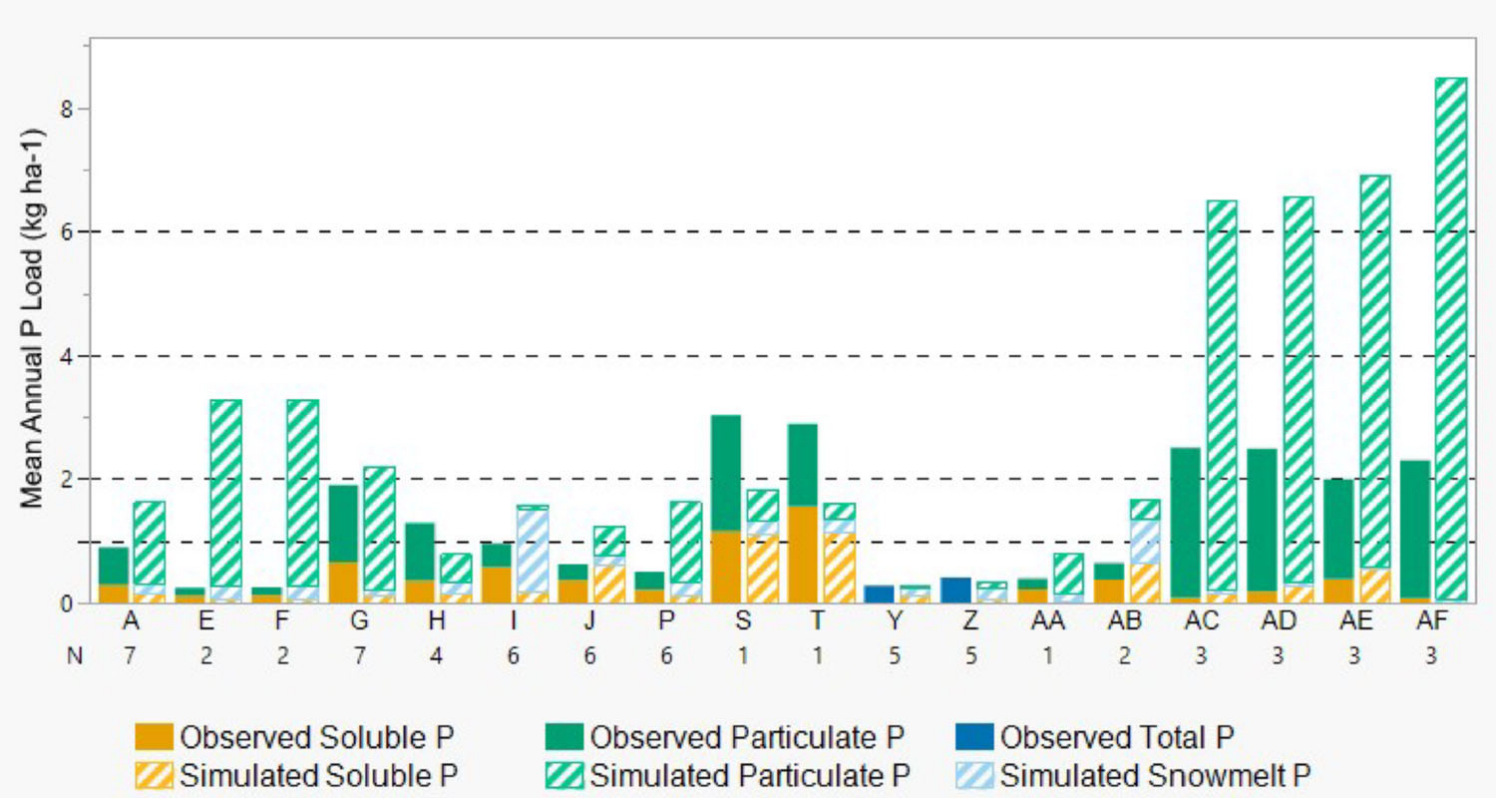
Comparison of Minnesota P Loss Index (MNPI)‐predicted annual average P loss to observed annual average P loss values (averages are across all available years by site). Dashed horizontal lines represent assigned risk categories based on modeled P loss (“very low risk of P loss”: 0–1 kg ha^−1^; “low risk of P loss”: 1–2 kg ha^−1^; “medium risk of P loss”: 2–4 kg ha^−1^; “high risk of P loss”: 4–6 kg ha^−1^; “very high risk of P loss”: 6 kg ha^−1^ or more). Observed total P is used for Sites Y and Z instead of particulate and soluble P because P fractions were not available for these two sites.

Although predicted and observed snowmelt P could not be directly evaluated, predicted snowmelt P generally represented a small fraction of predicted total P. When snowmelt P was a larger fraction of total P loss, it contributed to poor overall model performance, such as at sites I and AB (Figure 3). Phosphorus loss in snowmelt is governed not only by soil P concentration in the topsoil but also by antecedent soil moisture, surface cover, tillage intensity, snow water equivalent, and soil freeze‐thaw cycles (Kokulan et al., 2019; Wilson et al., 2019). Snowmelt runoff P can be modeled using complex hydrologic models such as ICECREAM and ICECREAMDB (e.g., Larsson et al., 2007; Radcliffe et al., 2015). However, the Wisconsin P index demonstrates that adequate snowmelt estimates can be developed without complex modeling. The snowmelt estimate in the Wisconsin P Index is based on observations of snowmelt runoff from 17 watersheds throughout the state and was found to perform well when compared against observed data (Good et al., 2012). In contrast, the MNPI bases its snowmelt estimate on a static value derived from only two field studies: (1) Hansen et al. (2000) (included in this study as sites E and F) and (2) Munyankusi (1999) (not included in this study because sites are located in Wisconsin on karst topography). Both of these studies are representative of conditions found within Southeastern Minnesota but are not representative of other parts of the state. It is unsurprising that MNPI provides a poor estimate of snowmelt P given the limited data used in generating its snowmelt estimates.

The sites with the highest disparity between MNPI‐predicted and observed average annual particulate P were also the sites without subsurface drainage systems (sites A, E, F, I, AB, AC, AD, AE, and AF). This may suggest that averaging MNPI results across site years better accounts for interannual variability at subsurface‐drained sites than at surface‐drained sites. However, no element used in the MNPI's prediction of particulate P explicitly considers the presence of subsurface drainage. There may be a confounding between sites that are not subsurface‐drained and the various landscape characteristics of sites that could affect the calculations (e.g., naturally well‐draining soil textures or steep slopes). As described above, particulate P prediction appears to be a key weakness of the model, and it may be linked to the tendency of RUSLE2 to overestimate particulate P.

### 3.3 Recommendations for model improvement and future study

The current amount of farm management data entered into the MNPI is comprehensive, encompassing a wide range of land management practices. Although the model did not perform well on an annual basis, its performance improved when considering higher intensity rainfall events and soluble P fractions averaged over time. Due to the level of detail from farm management records required to generate an MNPI prediction, we aimed to improve model predictions without requiring additional management inputs.

Two minor adjustments can be made to the MNPI to compensate for its tendency to overpredict P loss. First, the snowmelt P component can be removed from the calculation as it is not accurately calibrated for regions outside the southeast corner of Minnesota. This calculation was based on limited data and contributed to overprediction of P loss. Sharpley et al. (2017) urge caution when applying P indices outside the area in which they have been calibrated. While the snowmelt calculation may be accurate within Southeast Minnesota, there is currently no regional component to the MNPI to allow it to be added back in on a site‐by‐site basis. A future study on Minnesota's regional hydrogeomorphical characteristics, such as that conducted by Veith et al. (2017) for Pennsylvania, would be useful in drawing regional boundaries prior to adding a regional component to the MNPI. Second, the existing particulate P result can be multiplied by a weighting adjustment factor of 0.3 at sites without subsurface drainage. The goal of this adjustment is to address overprediction of soil erosion by RUSLE2. A similar approach adjusting weighting factors has been employed in other studies, such as the Kansas P Index (Sonmez et al., 2009). Modifications made to RUSLE2 to improve vegetative residue production as described in Dabney and Yoder (2012) are not present in the current MNPI. Replacing RUSLE2 calculations with an updated erosion model may be an approach that warrants further exploration in future updates to the MNPI. Applying these two adjustments brought the MNPI's prediction of annual average TP loss into the “sufficient” range (NSE = 0.51, RSR = 0.69, PBIAS = 7.3%). These proposed adjustments functionally calibrate the MNPI for the areas that have been monitored for this study (the southern half of Minnesota). Further adjustment may be needed as additional monitoring data becomes available.

This MNPI study collected as much data as available within the state of Minnesota at this time. Yet, gaps remain in the scope of geographic locations monitored and in the representation of different in‐field management practices that were conducted at these monitored sites. For instance, no sites located in the northern half of Minnesota were available for inclusion in this study due to a lack of data. Despite the well‐established value of monitoring data for making informed agronomic decisions (Daniels et al., 2018), Minnesota lags behind other Midwestern states in P loss data availability (Christianson et al., 2016). Improving availability of monitoring data to make informed agronomic decisions needs to be a priority for the health of Minnesota's freshwater resources.

Although we were able to find evidence of sufficient performance in this study, there is still room for improvement in MNPI performance. P site assessment tools have fundamental limitations because our understanding of and ability to model P fate and transport is still progressing (Sharpley et al., 2017). Incorporating separate subsurface and surface discharge components into MNPI calculations may further improve results. Several state P indices include explicit subsurface components of P loss (particularly states along the US East Coast and Eastern Corn Belt Shober et al. [2017]). A wealth of literature documents that, in addition to soil‐test P, additional factors such as P saturation, P application methods, surface runoff, and subsurface drainage all influence P loss (Sharpley & Withers, 1994; Sharpley et al., 2012). Further, surface and subsurface transport mechanisms act on in‐field P sources differently (King et al., 2015; Pease et al., 2018). It is not surprising that a one‐size‐fits‐all model of P “source” x “transport” does not adequately reflect these nuances.

## 4 CONCLUSIONS

Overall, the 2006 version of the MNPI is directionally correct (i.e., it can generally rank fields that are more at risk than others), but it is not magnitudinally correct. It should not be used as a field‐scale phosphorus loss prediction estimate due to its inconsistent performance on an annual basis. The 2006 MNPI provides an adequate prediction of average annual soluble P loss but tended to overpredict average annual particulate P loss. These limitations can be addressed by removing the snowmelt soluble P calculation and by adding a manual adjustment factor to RUSLE2 estimates for sites without subsurface drainage. Continuous re‐evaluation of the MNPI with new data as it becomes available will help ensure that the MNPI is properly assessing risk as our understanding of P loss in agricultural landscapes evolves. The decades‐long effort led by Sharpley to improve and iterate on P indices has allowed numerous states to improve their understanding of how local, field‐scale management affects P loss and water quality (Sharpley et al., 2012, 2017). It is critical that the MNPI continues to evolve alongside other state P indices. By following Sharpley's example, the scientific community will be well‐positioned to address the complex challenges of agricultural P.

## AUTHOR CONTRIBUTIONS

**Heidi Reitmeier**: Data curation; formal analysis; investigation; writing—original draft; writing—review and editing. **Lindsay Pease**: Conceptualization; funding acquisition; methodology; project administration; validation; writing—original draft; writing—review and editing. **Peyton Loss**: Formal analysis; writing—review and editing. **Tim Radatz**: Data curation.

## CONFLICT OF INTEREST STATEMENT

The authors declare no conflicts of interest.

## Supporting information



Additional information including the edge‐of‐field P loss data used to evaluate the MNPI, data collection methodology, and a summary the of field and management data used in MNPI predictions are available in the supplemental materials. The supplemental materials also include additional figures showing the comparison between modeled and observed P loss for both particulate P and soluble P, P loss for site years with at least one rainfall event greater than 51 mm in a 24‐hr period, and P loss for site years with at least one rainfall event greater than 104 mm in a 24‐hr period.

Supporting Information
