# Supplementary material for: Evaluation of Minnesota Phosphorus Loss Index performance
Source: J Environ Qual. 2024 Oct 8;54(4):827–37. doi: 10.1002/jeq2.20635 (PMC12265854; doi:10.1002/jeq2.20635)
Supplement: Supplementary file 1 — Additional information including the edge‐of‐field P loss data used to evaluate the MNPI, data collection methodology, and a summary the of field and management data used in MNPI predictions are available in the supplemental materials. The supplemental materials also include additional figures showing the comparison between modeled and observed P loss for both particulate P and soluble P, P loss for site years with at least one rainfall event greater than 51 mm in a 24‐hr period, and P loss for site years with at least one rainfall event greater than 104 mm in a 24‐hr period. [file JEQ2-54-827-s001.docx]

**
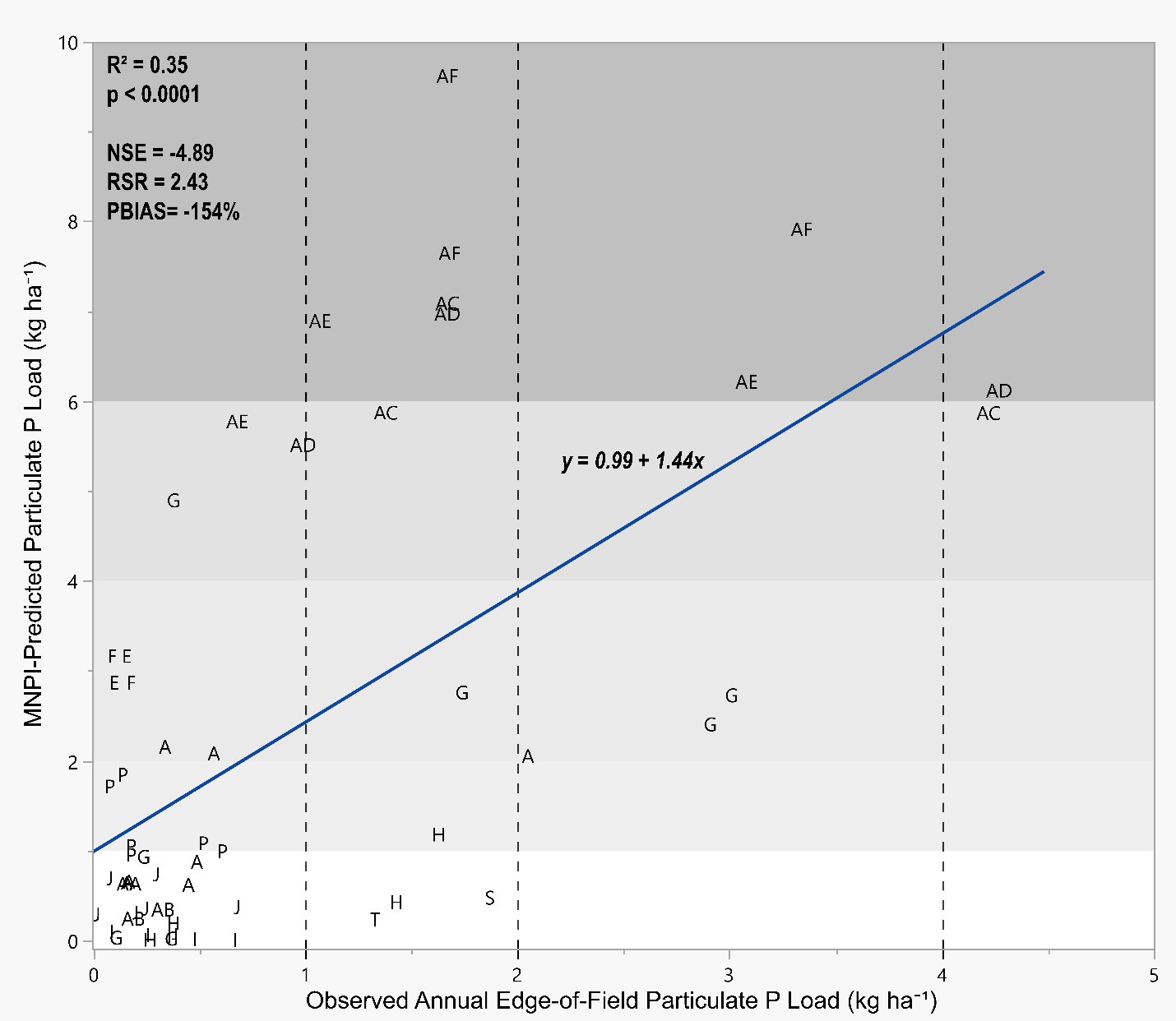
**

**Supplemental Figure S1: Relationship between MNPI-predicted and observed annual soluble P loss. Shaded areas represent assigned risk categories based on modeled P loss (“very low risk of P loss”: 0-1 kg ha^-1^; “low risk of P loss”: 1-2 kg ha^-1^; “medium risk of P loss”: 2-4 kg ha^-1^; “high risk of P loss”: 4-6 kg ha^-1^; “very high risk of P loss”: 6 kg ha^-1^ or more). Dashed vertical lines represent equivalent assigned risk category based on observed data. “NSE” is to Nash-Sutcliffe Efficiency, “RSR” is root mean standard deviation ratio, and “PBIAS” is percent bias as defined in Moriasi et al (2007).**

**
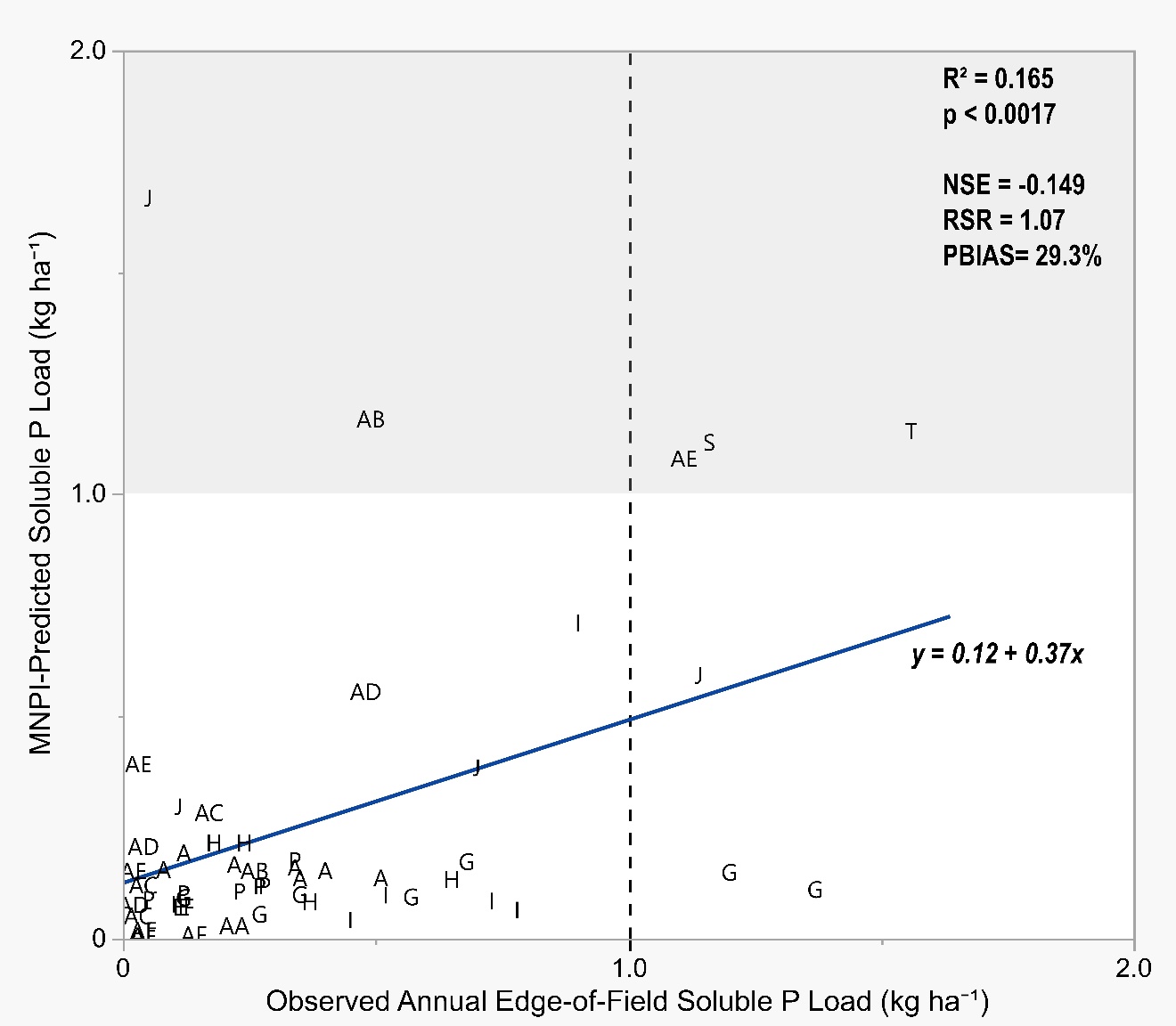
**

**Supplemental Figure S2: Relationship between MNPI-predicted and observed annual soluble P loss. Shaded areas represent assigned risk categories based on modeled P loss (“very low risk of P loss”: 0 to 1 kg ha^-1^; “low risk of P loss”: 1 to 2 kg ha^-1^; “medium risk of P loss”: 2 to 4 kg ha^-1^; “high risk of P loss”: 4 to 6 kg ha^-1^; “very high risk of P loss”: 6 kg ha^-1^ or more). Dashed vertical lines represent equivalent assigned risk category based on observed data. “NSE” is to Nash-Sutcliffe Efficiency, “RSR” is root mean standard deviation ratio, and “PBIAS” is percent bias as defined in Moriasi et al (2007).**


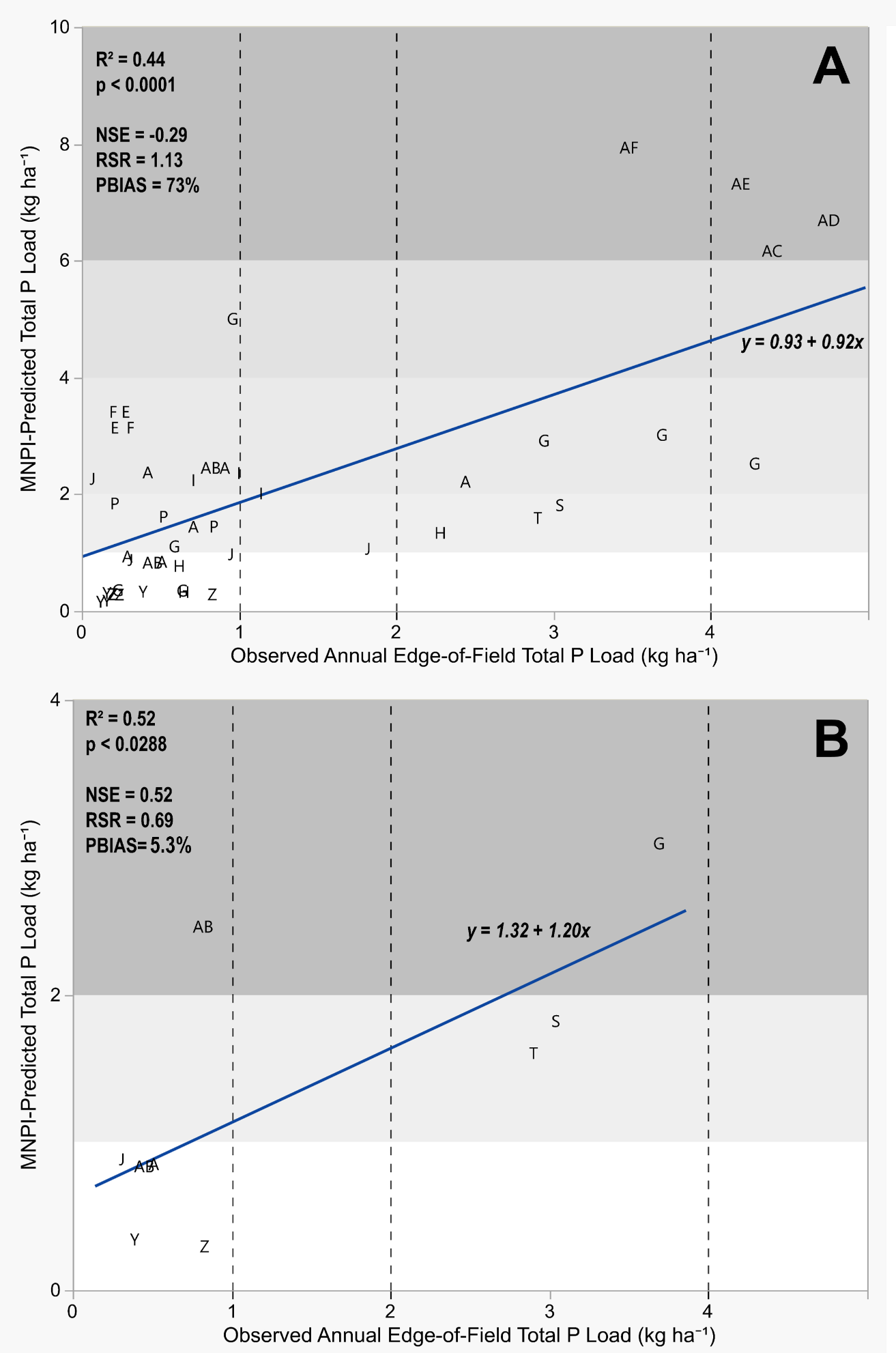


**Supplemental Figure S3: Relationship between MNPI-predicted and observed annual total P loss for site years with at least one rainfall event greater than (A) 51 mm in a 24-hr period (n = 46 site-years or (B) 104 mm in a 24-hr period (n = 9 site-years). Shaded areas represent assigned risk categories based on modeled P loss (“very low risk of P loss”: 0-1 kg ha^-1^; “low risk of P loss”: 1-2 kg ha^-1^; “medium risk of P loss”: 2-4 kg ha^-1^; “high risk of P loss”: 4-6 kg ha^-1^; “very high risk of P loss”: 6 kg ha^-1^ or more). Dashed vertical lines represent equivalent assigned risk category based on observed data. “NSE” is to Nash-Sutcliffe Efficiency, “RSR” is root mean standard deviation ratio, and “PBIAS” is percent bias as defined in Moriasi et al (2007).**
